# MnSOD Lysine 68 acetylation leads to cisplatin and doxorubicin resistance due to aberrant mitochondrial metabolism

**DOI:** 10.7150/ijbs.51184

**Published:** 2021-03-19

**Authors:** Yucheng Gao, Yueming Zhu, Elizabeth L. Tran, Valerie Tokars, Angela E. Dean, Songhua Quan, David Gius

**Affiliations:** 1 Department of Radiation Oncology and Northwestern University, Chicago, IL, USA.; 2 Driskill Graduate Program in Life Sciences, Northwestern University, Chicago, IL, USA.; 3 Department of Pharmacology, Northwestern University.; 4 Division of Nutritional Sciences, University of Illinois at Urbana-Champaign.; 5 Department of Pharmacology, Robert H. Lurie Cancer Center, Northwestern University Feinberg School of Medicine, Chicago, IL, USA.; 6 Department of Radiation Oncology, Joe R. & Teresa Lozano Long School of Medicine, University of Texas Health San Antonio, TX, USA.

**Keywords:** Sirtuins, SIRT3, MnSOD, SOD2, acetylation, acetylome, lysine 68, mitochondria, metabolism, carcinogenesis, aging, signaling

## Abstract

Manganese superoxide dismutase (MnSOD) acetylation (Ac) has been shown to be a key post-translational modification important in the regulation of detoxification activity in various disease models. We have previously demonstrated that MnSOD lysine-68 (K68) acetylation (K68-Ac) leads to a change in function from a superoxide-scavenging homotetramer to a peroxidase-directed monomer. Here, we found that estrogen receptor positive (ER+) breast cancer cell lines (MCF7 and T47D), selected for continuous growth in cisplatin (CDDP) and doxorubicin (DXR), exhibited an increase in MnSOD-K68-Ac. In addition, MnSOD-K68-Ac, as modeled by the expression of a validated acetylation mimic mutant gene (*MnSOD^K68Q^*), also led to therapy resistance to CDDP and DXR, altered mitochondrial structure and morphology, and aberrant cellular metabolism. *MnSOD^K68Q^* expression in mouse embryo fibroblasts (MEFs) induced an *in vitro* transformation permissive phenotype. Computerized molecular protein dynamics analysis of both MnSOD-K68-Ac and MnSOD-K68Q exhibited a significant change in charge distribution along the α1 and α2 helices, directly adjacent to the Mn^2+^ binding site, implying that this decrease in surface charge destabilizes tetrameric MnSOD, leading to an enrichment of the monomer. Finally, monomeric MnSOD, as modeled by amber codon substitution to generate MnSOD-K68-Ac or MnSOD-K68Q expression in mammalian cells, appeared to incorporate Fe to maximally induce its peroxidase activity. In summary, these findings may explain the mechanism behind the observed structural and functional change of MnSOD-K68-Ac.

## Introduction

It is well-established that the detoxification activity of antioxidant enzymes is dysregulated in tumors 1, 2, implying that a loss of reactive oxygen species (ROS) buffer or detoxification activity corresponds to a more oncogenic and/or therapy-resistant phenotypes 1-3. In particular, Manganese superoxide dismutase (MnSOD) is an evolutionarily conserved protein that functions as a primary mitochondrial superoxide scavenger and is part of the superoxide dismutase family (SOD1-3), which catalyzes the conversion of superoxide molecules (O_2_^-^) produced from mitochondrial respiration to H_2_O_2_ 4-7 and diatomic oxygen 8, 9. The homotetrameric MnSOD, with four identical subunits each harboring a Mn^2+^ atom 10, is encoded in the nucleus and then translocated to the mitochondrial matrix where the targeting signal is cleaved post-translationally 11. MnSOD has traditionally been thought of as a mitochondrial fidelity protein or a tumor suppressor (TS), and MnSOD mutations and decreased protein levels are associated with idiopathic cardiomyopathy (IDC), premature aging, motor neuron disease, and importantly, oncogenicity and resistance to anticancer agents 12. Moreover, MnSOD post-translational modifications, especially acetylation/deacetylation regulated by SIRT3, which has been recognized as the primary mitochondrial deacetylase 13, 14, have been proven to regulate MnSOD activity and function in several different cellular pathways 15-19.

In this regard, it has been known that *Sirt3* silencing results in an increase in MnSOD acetylation, thus, leading to aberrant ROS accumulation upon stress, which induces genome instability and dysregulated cellular and mitochondrial bioenergetics 20-24. Genetic deletion of *Sirt3* leads to a tumor-permissive phenotype, as shown using both *in vivo* models and* in vitro* assays that measure transformation and/or tumorigenesis, suggesting that SIRT3 functions as a mitochondrial TS. Furthermore, female mice lacking *Sirt3* appear to spontaneously develop estrogen receptor-positive (ER+), high Ki-67, poorly differentiated, mammary gland tumors that seem to be similar, as analyzed by immunohistochemistry, to human luminal B breast malignancies, which account for the most death cases among all breast cancer patients in the United States 20.

Several MnSOD lysines have been identified as biochemical and/or physiological SIRT3 deacetylation targets - K53, K89 25, 26, K68 27-29, and K122 19, 23 - using different direct or indirect experimental techniques, including site-directed mutagenesis, physical lysine acetylation, and acetyl-lysine specific monoclonal antibodies. However, the specific cell biological, biochemical, and/or physiological significance and cellular context for the role of each of these lysines, as well as the underlying molecular mechanism by which they regulate MnSOD detoxification activity and mitochondrial metabolism, remains to be fully determined. In this regard, **(i)** Dr. Danica Chen's lab has published two elegant manuscripts 25, 26 convincingly showing that MnSOD-K53-Ac and K89-Ac direct enzymatic and biological function; and **(ii)** Dr. Qing Xia's lab 17 published that “K68 site is the most important acetylation site contributing to SOD2 activation”. Based on recent data from our laboratory 30, 31, as well as that published by others 27-29, we began to explore how changes in protein dynamics, due to K68-Ac, affect MnSOD activity.

MnSOD has been suggested to be a TS protein, both *in vitro* and *in vivo* 2, 32, as well as in human tumor samples 33. However, there are correlative findings in human tumor samples suggesting that while MnSOD may function as a TS during the early stages of tumor initiation, once tumorigenesis progresses MnSOD levels positively correlate with more aggressive human tumors 34. Recently, our lab discovered that MnSOD-K68-Ac presents as a monomeric form and exhibits a peroxidase-inducing, tamoxifen-resistant, tumor-permissive phenotype 30. Nonetheless, the mechanism behind these observations, and whether this drug resistance could be applied in a broader context, is still unclear.

In this paper, we showed that dysregulation and/or disruption of the physiological MnSOD-K68-Ac axis can lead to a chemotherapy resistant phenotype in ER+ breast cancer. In addition, because of the pivotal role of MnSOD in mitochondria, we hypothesized that the expression of the MnSOD acetylation mimetic might direct the dysregulation of mitochondrial morphology and ultrastructure, as well as disrupt mitochondrial metabolism. Finally, we presented data discussing how metal cooperation within MnSOD would affect its peroxidase activity and function. Overall, we hope to expand the knowledge of MnSOD-K68-Ac's role as a critical regulator in cancer models and as a potential therapeutic target in the future.

## Methods

### Cell lines

The wild-type ER+ MCF7 human breast cancer cells and immortalized MnSOD^-/-^ mouse embryonic fibroblast cells (MEF) were cultured at 37 °C with 5% CO_2_ in regular growth medium, which is composed of Dulbecco's Modified Eagle's Medium (DMEM, Gibco) with 10% fetal bovine serum (FBS; Sigma) and 1% Antibiotic Antimycotic solution (Sigma). Primary MEFs were isolated from isogenic mouse embryos (E13.5) and cultured at 37 °C with 5% CO_2_ and 6% oxygen. Lenti-virally infected MCF7 and MEFs were grown in media with 1 μg/ml puromycin. Cisplatin and doxorubicin resistant MCF7 cells were treated for over 3 months to establish permanent cell lines. All experiments were done using exponentially growing cells at 50%-70% confluence.

### Lentiviral infection

Human Lenti-MnSOD plasmid was used for site-directed mutagenesis where lysine at location 68 is mutated to either arginine (deacetylation mimetic) or glutamine (acetylation mimetic) (Bioinnovatise). HEK 293T cells were transfected with 5 μg DNA of interest, 5 μg psPAX2 packaging plasmid, and 300 ng VSV.G envelope plasmid. Fresh medium was added after overnight incubation and viral supernatant was collected after an additional 48 hours and filtered using a 0.45 μm filter (Corning). MCF7 and MEFs were lenti-virally infected at 40% confluence with 10 μg/ml polybrene for 72 hours. Cells were subsequently recovered with regular medium for 24 hours and then selected with 1μg/ml puromycin.

### Western blot

Cell pellets were collected, lysed with radioimmunoprecipitation assay (RIPA) buffer with protease inhibitor, and centrifuged at 10,000 g for 20 min. Protein concentration were quantified by the Bradford protein assay (Bio-Rad). Proteins samples were run and separated using 4-12% NuPAGE Bis-Tris polyacrylamide (Invitrogen) and 1X MOPS SDS running buffer (Invitrogen, 20X). The gels were transferred to nitrocellulose membranes. The membranes were then blocked with 5% milk for 1 hour at room temperature and immunoblotted with one of the following primary antibodies: β-tubulin (Proteintech, 1;10,000 dilution), α-actin (Proteintech, 1:10.000 dilution), MnSOD (Millipore, 1;1,000 dilution), MnSOD-K68-Ac (Abcam, 1;1,000 dilution) overnight. The membranes were subsequently blotted with either mouse or rabbit secondary antibody and visualized using enhanced chemiluminescence (ECL) development.

### Clonogenic cell survival assay

To evaluate clonogenic cell survival at low density, 500 exponentially growing cells were plated in triplicate in 6-well plates using serial dilution, and the growth of the cells was examined throughout 14 days in regular growth medium. Cells were fixed with 70% ethanol for 5 minutes and then stained with 0.5% crystal violet (in 25% methanol) for 20 minutes. Photos of stained plates were taken, and colonies of more than 50 cells were counted and used to calculate clonogenic survival.

### Soft agar colony formation assay

Ten thousand cells were plated in triplicate on 0.3% agar in growth medium 1X over a 0.6% base agar foundation layer in growth medium 1X (growth medium 2X consisted of DMEM supplemented with 20% FBS, 2% penicillin-streptomycin, 1% 2.5 M glucose and 2% GlutaMax 100X). The size of colonies was monitored over a period of 3 weeks, and by the end of 3 weeks, colony growth was visualized via microscope, and images were acquired.

### MTT cell proliferation assay

Cell proliferation was measured using MTT proliferation assay kit (ab211091). 10,000 exponentially growing cells were plated in regular growth medium per well into 96-well plates in triplicate. Cells were treated with specified drugs after overnight and incubated for 48 hours. Treatment media was then discarded and a mixture of 50 μl MTT reagent and 50 μl serum-free media was added into each well and incubated for 3 hours at 37ºC. 150 μl of MTT solvent was then added, and the plate was shaken for 15 minutes avoiding light. The absorbance was read at OD=590 nm and used to evaluate cell proliferation.

### Transmission Electron Microscopy and Immunogold Electron Microscopy

Post-imbedding procedures were carried out at Northwestern University's Center for Advanced Microscopy. In brief, cells of interest were collected, rinsed with PBS (1X) once, resuspended in 500 μl fixative (2% paraformaldehyde + 2.5% glutaraldehyde in 0.1 M cacodylate buffer) and fixed at room temperature for an hour. After post-fixation with 3% uranyl acetate, cells were dehydrated in series of ethanol, embedded in LRWhite resin and polymerized for 48 hours at 50 ºC. Then ultrathin sections were made using UC7 ultramicrotome (Leica Microsystems) and placed on nickel grids. Immunostaining grids were incubated with blocking solution (50 mM glycine for 15 min) and then with primary MnSOD antibody for 3-4 hours at room temperature. After several washes with PBS with 0.1% BSA, sections were incubated with goat anti-Rabbit secondary antibody conjugated with 12 nm gold (Jackson Immunoresearch) in 1:100 dilution overnight at 4 ºC. After washing with PBS and post-fixing with 1% glutaraldehyde in water, sections were contrasted with 4% uranyl acetate and Reynolds's lead citrate. Samples were imaged using a FEI Tecnai Spirit G2 transmission electron microscope (FEI Company, Hillsboro, OR) operated at 80 kV. Images were captured by an Eagle 4k HR 200kV CCD camera.

For regular transmission electron microscopy, cells were fixed in the same fixative as described above and were prepared for 100 μm thick sections and then embedded based on standard protocol by the Northwestern University's Center for Advanced Microscopy. The sample sections were imaged as described above.

### Incorporation and isolation of N-(ε)-Acetyl-Lysine into MnSOD-K68

BL21 (DE3) pMAGIC bacteria were co-transformed with pEVOL-AcKRS, which expresses an acetyl-lysyl-tRNA synthetase/tRNA^CUA^ pair from *M. barkeri*, and pET21a-MnSOD^K68TAG^, which expresses a site-specific mutation that allows incorporation of N-(ϵ)-acetyl-l-lysine (AcK) into K68. BL21(DE3) pMAGIC cells were transformed with pEVOL-AcKRS and pET21a-MnSODK68TAG were cultured in 3 ml of sterile LB media with 300 μg/ml ampicillin, 50 μg/ml kanamycin and 50 μg/ml chloramphenicol, and 1ml of the culture was then cultured in 100ml of LB media with 300 μg/ml ampicillin, 50μg/ml kanamycin and 50 μg/ml chloramphenicol overnight (200 rpm, 37 °C). 1 ml of the overnight culture was inoculated in 100 ml of LB media with the same antibiotic concentration and shaken at 220 rpm, 37 °C until OD = 600 nm reaches 0.6. Bacterial culture was induced with 0.4 mM IPTG, nicotinamide, arabinose and N-acetyl lysine and shaken overnight at 180 rpm, room temperature.

Protein was lysed in buffer (20 mM imidazole, 50 mM Tris-HCl, 200 mM NaCl, pH=8) with 1.5 mg/ml PMSF and 1 mg/ml lysozyme. Lysates were incubated on ice for 10 minutes, sonicated for 20 minutes (10s on, 5s off, 50% amplitude), and centrifuged for supernatant (13,000 g, 30 min). 0.2 mL Ni2+ NTA beads were added to the collected supernatant and rotated for 1 hour at 4 °C. Protein was filtered via passing the supernatant through Probond Purification System filter column. The column was washed three times using the lysing buffer. Then the protein was eluted with elution buffer (250 mM imidazole, 50 mM Tris-HCl, 200 mM NaCl, pH=8) and quantified for further experiments.

### Peroxidase activity assay

The peroxidase enzymatic activities isolated or pulled-down proteins were determined by using pyrogallol as the substrate. The reaction mixture was composed of 14 mM potassium phosphate (Sigma), 0.027% (v/v) hydrogen peroxide (Sigma), and 0.5% (w/v) pyrogallol (Sigma). Samples were mixed well and incubated for 10 minutes at room temperature, and then read at OD=420 nm. Every 3 minutes, the increase in the reading was recorded. The ΔA420/20 sec was obtained using the maximum linear rate or 3-minute interval for controls and test samples. The peroxidase activity was calculated using the following equation: Units/mL = [(ΔA420/20 sec Test Sample - ΔA420/20 sec Blank)(reaction volume)(dilution factor)]/[(12)(0.1)].

### Statistical analysis

Statistical analysis was performed using GraphPad Prism for Windows (GraphPad Software, San Diego, CA). Data were expressed as mean SEM unless otherwise specified. One-way ANOVA analysis with Tukey's post-analysis was used to study the differences among three or more means. Significance was determined at p<0.05 and the 95% confidence interval.

## Results

### Cisplatin and doxorubicin-resistant breast cancer cells exhibit an increase in MnSOD-K68-Ac

We have previously shown that MnSOD-K68-Ac is enriched in a subgroup of luminal B breast malignancies 30, which commonly recur with endocrine therapy, and is a mitochondrial based signaling network for the development of tamoxifen resistance (TamR), as determined using breast cancer tissue culture cells. Here, we further explored whether this resistance phenotype could be extended to a broader application in other standard treatments in women with luminal B breast cancer, including cisplatin (CDDP) and doxorubicin (DXR). To address this, we used a standard method to select tissue culture for resistance to anticancer agents in both MCF7 and T47D, two established ER+ breast cancer cell lines 35.

**Figure 1:**
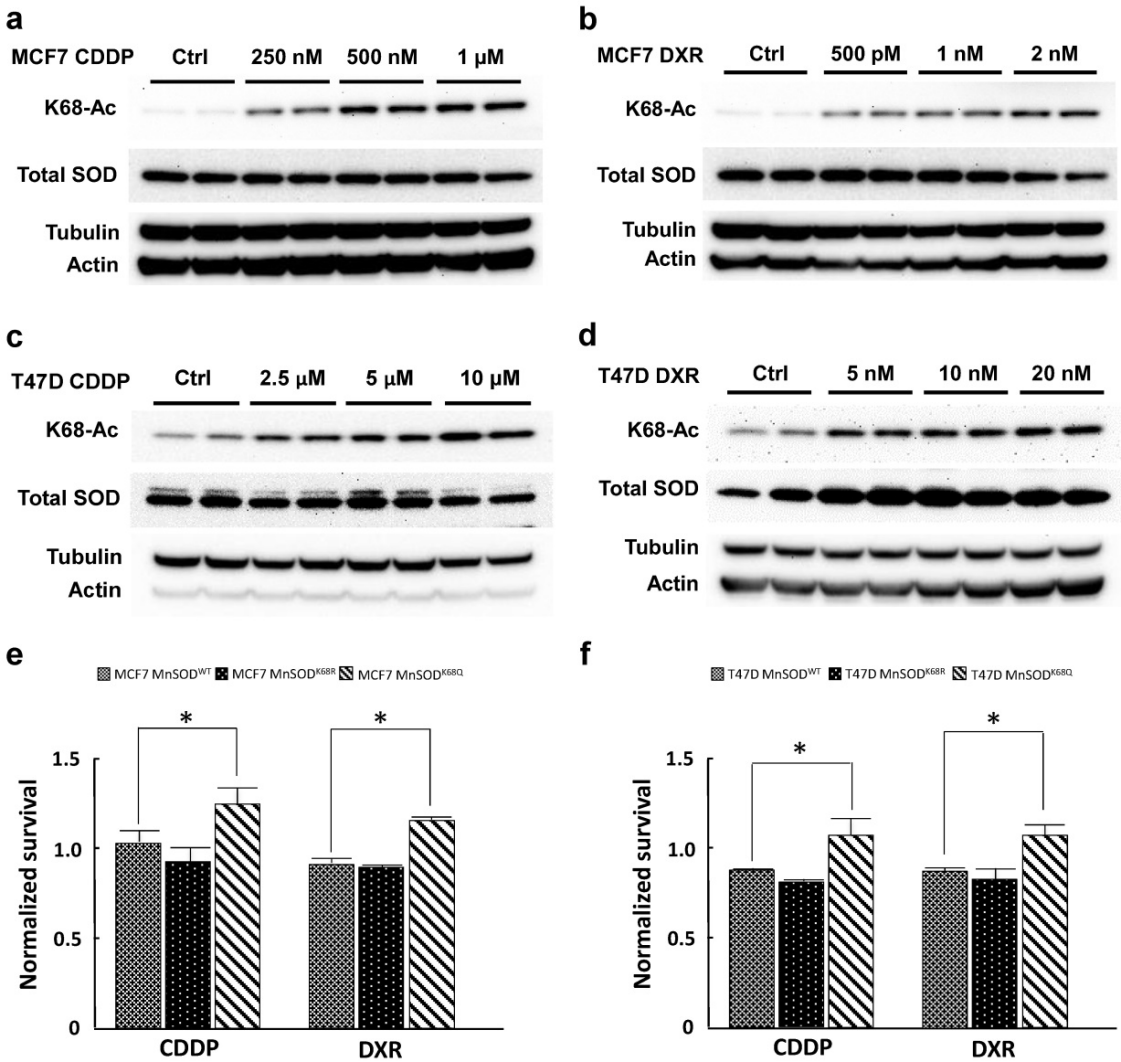
** Cisplatin and doxorubicin-resistant breast cancer cells exhibit an increase in MnSOD-Ac. (a,b)** Cell lysates of 250nM, 500nM, 1µM CDDP-resistant and 500pM, 1nM and 2nM DXR-resistant MCF7 cells (cultured in drug-containing media for 3 months) were collected and immunoblotted for MnSOD-K68-Ac, MnSOD, actin and tubulin. **(c,d)** Cell lysates of 2.5 µM, 5 µM, 10 µM CDDP-resistant and 5 nM, 10 nM, 20 nM DXR-resistant T47D cells (also cultured for 3 months in drug-containing media) were collected and immunoblotted for MnSOD-K68-Ac, MnSOD, actin and tubulin. **(e,f)** 10,000 MCF7 or T47D cells overexpressed with empty vector, *MnSOD^WT^, MnSOD^K68R^* or* MnSOD^K68Q^* were plated in 96 well plate and treated with CDDP (1 µM for MCF7 and 10 µM for T47D) or DXR (1 nM for MCF7 and 10 nM for T47D) the next day. After 48 hours, MTT assay was conducted to determine the cell viability after chemotherapy drug treatment. The results of cells infected with *MnSOD^WT^, MnSOD^K68R^* or* MnSOD^K68Q^* were normalized to those of cells infected with empty vector. All experiments were done in triplicates. Error bar represents ±1 SEM. *p<0.05.

MCF7 and T47D cells were selected for resistance with three different doses of CDDP (250 nM, 500 nM, 1 µM; and 2.5 µM, 5 µM, 10 µM, respectively) and DXR (500 pM, 1 nM, 2 nM; and 5 nM, 10 nM, 20 nM) for 3 months. Both CDDP-resistant and DXR-resistant MCF7 and T47D cells displayed a dose-dependent increase in MnSOD-K68-Ac (Fig. 1a-d) without changes in total MnSOD protein levels. To study the effects of K68 acetylation on drug resistance, K68 acetylation and deacetylation mimic mutants (*MnSOD^K68Q^* and *MnSOD^K68R^*, respectively) were made where the substitution of a lysine (K) with a glutamine (Q) mimics a constitutively acetylated amino acid state, while the substitution with an arginine (R) mimics constitutive deacetylation 19,30, 31. To further confirm our observation, we infected MCF7 and T47D cells with either *MnSOD^WT^*,* MnSOD^K68R^* or *MnSOD^K68Q^*, and MnSOD exogenous protein expression was very similar, as verified via western blot (Fig. S1), while basal expression of endogenous MnSOD was quite minimal. These results were normalized to MCF7 and T47D cells infected with empty vector and showed that cells overexpressing *MnSOD^K68Q^* exhibited higher resistance to the short-term treatment (48 hours) of the highest dose of CDDP (1 µM for MCF7 and 10 µM for T47D) and DXR (2 nM for MCF7 and 20nM for T47D) (Fig. 1e, f). Hence, these results suggested a role for the disruption of MnSOD biology, through dysregulated MnSOD-Ac, and a potential pan resistant (PanR) tumor cell phenotype in MCF7 and T47D breast cancer cells.

### Primary MEFs (pMEFs) infected with MnSOD^K68Q^ exhibit a more transformative phenotype

**Figure 2:**
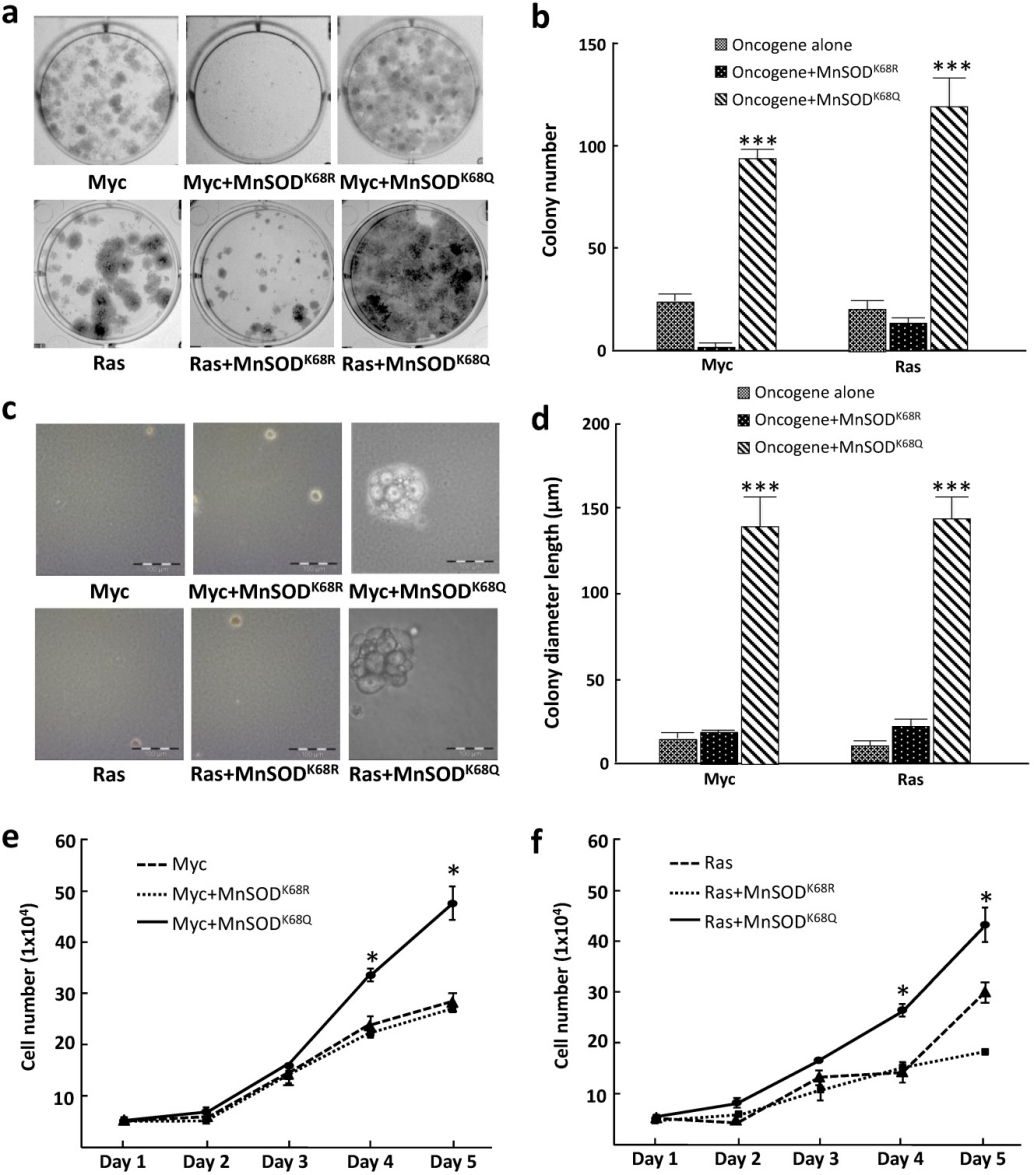
** Immortalized MEFs expressing *MnSOD^K68Q^* exhibit a more transformative phenotype**. **(a)** MEFs infected with lentivirus expressing *MnSOD^K68Q^*, and either *Myc* (top panels) or *Ras* (bottom panels) were puromycin selected and 500 cells were plated onto a 6-well plate. Cells were cultured for three weeks and plates were stained with crystal violet and colonies greater than 50 cells were counted. **(b)** Quantification of the average for colonies greater than 50 cells per 60 mm plate are shown in the bar graphs for the experiments in 2a. **(c)** 10,000 MEFs described above were plated in soft agar for 3 weeks and pictures were taken at the end of the experiment. **(d)** Quantification of the colony diameter are shown in the bar graph for figure 2b. **(e,f)** Growth of cell number over 5 days of the MEFs expressing either *Myc* (2e) or *Ras* (2f) described were counted as shown. Representative images are shown. All experiments were done in triplicates. Error bar represents ±1 SEM. *p<0.05, **p<0.01, and ***p<0.001.

To further examine how MnSOD-K68 acetylation affects the *in vitro* cell phenotype, we performed colony formation assays where tumor cell growth was determined when plated at low density, a measure of tumor cell proliferative capacity, i.e., 500 cells plated in 6-well plates, and stained with crystal violet after three weeks of culture (colonies required more than 50 cells). These experiments showed that pMEF cells infected with *MnSOD^K68Q^* (K68-Ac mimic mutant), as well as *Myc* or *Ras* exhibited an increase in colony formation (Fig. 2a, right upper/lower panels) compared to cells expressing the deacetylation mimic mutant (*MnSOD^K68R^*) and *Myc*, *Ras*, or *Myc*/*Ras* alone (Fig. 2a), and the number of colonies was quantified (Fig. 2b). To determine the anchor-independent growth of these three cell lines, 10,000 cells were plated in soft agar and the size of the colonies formed was observed and measured after three weeks. Again, colonies formed by pMEFs expressing *MnSOD^K68Q^* with one oncogene (Fig. 2c, right upper/lower panels) were significantly larger than those expressing *MnSOD^K68R^* with one oncogene (Fig. 2c, middle upper/lower panels) or *Myc*/*Ras* alone (Fig. 2c, left upper and lower panels). The diameter of the colonies was quantified (Fig. 2d). A growth curve over 5 days for the cell lines mentioned above was monitored and similarly, MEFs infected with *MnSOD^K68Q^* with *Myc* or *Ras* exhibited the fastest growth rate (Fig. 2e, f), which corresponds to the results from the colony formation and soft agar assays. These data further confirmed that the expression of *MnSOD^K68Q^*, the acetylation mimetic, leads to a transformative phenotype *in vitro*.

### Cells expressing MnSOD^K68Q^ display disrupted mitochondrial morphology and ultrastructure

**Figure 3:**
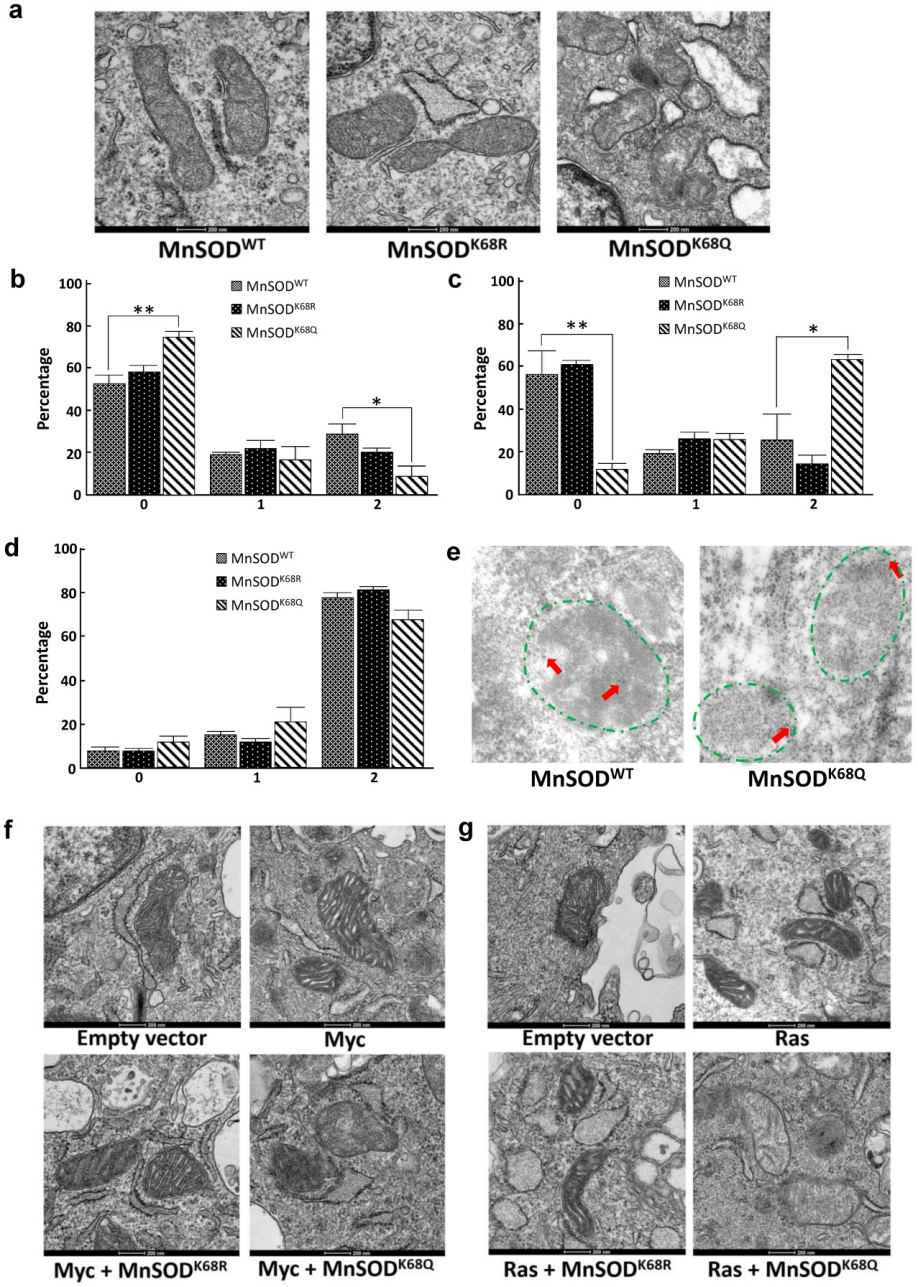
** MnSOD^K68Q^ expressed in MEFs shows dysregulated mitochondrial structure and localization. (a)** Cell pellets of SOD2^-/-^ MEFs overexpressing *MnSOD^WT^*, *MnSOD^K68R^*, and *MnSOD^K68Q^* were rinsed with PBS and fixed in 2% paraformaldehyde (PFA) + 2.5% glutaraldehyde (GA) in 0.1M cacodylate buffer and further processed at Northwestern University's Center for Advanced Microscopy. Sample slides were imaged via electron microscopy. Representative images are shown; **(b)** Mitochondrial size was quantified (0=small<0.7um, 1=intermediate, 2=large>0.8um) and visualized via bar graph; **(c)** Cristae structure was quantified and visualized (0=clear, 1=intermediate, 2=destroyed);** (d)** Quantification of mitochondrial electron density (0=low, 1=intermediate, 2= high) was visualized via bar graph;** (e)** SOD2^-/-^ MEFs overexpressing *MnSOD^WT^* and *MnSOD^K68Q^* were fixed with 2% PFA + 0.5% GA in 0.1M cacodylate buffer and further processed at Northwestern University's Center for Advanced Microscopy. Samples were stained with rabbit anti-MnSOD (Millipore) and donkey anti-rabbit IgG conjugated to 10-nm gold particles and images were taken using electron microscopy. Mitochondria (area surround with dashed green lines) and gold particles (red arrows) were identified by its shape, density, cristae structure, the overall number of the organelle with the same shape and expert opinion from the microscopy center. **(f, g)** MEFs infected with empty vector, *Myc* (Fig. 3f) or *Ras* (Fig. 3g), *MnSOD^K68R^* with *Myc* or *Ras*, *MnSOD^K68Q^* with *Myc* or *Ras*, were fixed and processed as described above and were imaged using electron microscopy. Representative images are shown. All experiments were done in triplicates. Error bar represents ±1 SEM. *p<0.05, **p<0.01.

Since MnSOD is a critical protein located inside the mitochondria, which plays a central role in directing ROS detoxification and maintaining cellular and mitochondrial metabolism, we aim to further explore whether the acetylation status of MnSOD would affect the structure of mitochondria via transmission electron microscopy (TEM). Our previous research has shown that enforced *MnSOD^K68Q^* overexpression leads to an *in vitro* transformation permissive phenotype; however, these experiments were complex due to the presence of endogenous wild-type MnSOD. To eliminate the effect of endogenous MnSOD expression and more rigorously examine the function of MnSOD^K68Q^, immortalized MnSOD^-/-^ MEFs were infected with either *MnSOD^WT^*, *MnSOD^K68R^*, or *MnSOD^K68Q^* and permanent cell lines were generated. Mitochondria have an intact double membrane structure, with its inner membrane interfolding into clear cristae compartments while its outer membrane tends to be stiff and more cell membrane-like. In this regard, MnSOD^-/-^ MEFs overexpressed with *MnSOD^K68Q^* (acetylation mimic mutant) displayed loss of double-membrane with a disarrayed cristae arrangement, as compared to SOD^-/-^ cells overexpressed with *MnSOD^WT^* and *MnSOD^K68R^* (Fig. 3a) 36-39. The difference in morphology and ultrastructure was further quantified according to a 0-2 scoring system published by the Yasuda group 38, 40. Mitochondria size was scored based on the longest diameter (0=small < 0.7 µm; 1=intermediate; 2=large >0.8 µm), and MnSOD^K68Q^ MEFs displayed significantly larger distribution of small mitochondria and fewer populations of large mitochondria compared to both MnSOD^WT^ and MnSOD^K68R^ MEFs (Fig. 3b). Cristae structure was also evaluated based on how intact the compartmentalization remained (0=clear, 1=intermediate, 2=destroyed). Importantly, it was observed that most mitochondria from the MEFs expressing *MnSOD^K68Q^* had disrupted inner cristae alignment (Fig. 3c), but their cell electron density remained normal (Fig. 3d), in comparison to MnSOD^-/-^ MEFs expressing either *MnSOD^WT^* or *MnSOD^K68R^*.

To further investigate whether *MnSOD^K68Q^* expression would affect the localization inside the mitochondria, immunogold electron microscopy was conducted where the location of the gold particles bound to the protein could be visualized (Fig. 3e, Fig. S2a). The identification of the mitochondria (within the area of green dashed lines) and the gold particles (red arrows), with the assistance of expert opinions from the microscopy center, was based on mitochondrial features, such as shape, density, and cristae structure, as well as the gold particles' size and density. Through the TEM microscope and the subsequent quantification, we found that wild-type MnSOD in SOD^-/-^ MEFs was located in the inner mitochondrial area (Fig. S2b); while in contrast, MnSOD-K68Q was found on the rim of multiple mitochondria, suggesting a potential relation between MnSOD mitochondrial localization and its acetylation status.

Similar experiments were also done in MEFs immortalized by co-infection with lenti-*MnSOD^K68Q^*, which we have indicated above functions as a tumor promoter/gene, and a second oncogene, i.e., *Myc* (Fig. 3f) or *Ras* (Fig. 3g). These TEM showed altered/aberrant mitochondrial ultrastructure compared to control MEFs infected with empty vector, *Myc*, or *Ras* alone. There was also a significant decrease in the observed mitochondria size in *MnSOD^K68Q^* infected MEFs (Fig. S3a, b) and an increase in the number of significantly damaged mitochondria (Fig. S3c, d). Interestingly, no difference was detected in electron density between MEFs expressing MnSOD^K68Q^ and control groups (Fig. S3e, f); and a similar but less obvious observation about cristae structure could also be made between MCF7 cells overexpressed with *MnSOD^WT^* and *MnSOD^K68Q^* (Fig. S4).

### Acetylation status of MnSOD-K68 directs the reprogramming of mitochondrial metabolism

**Figure 4:**
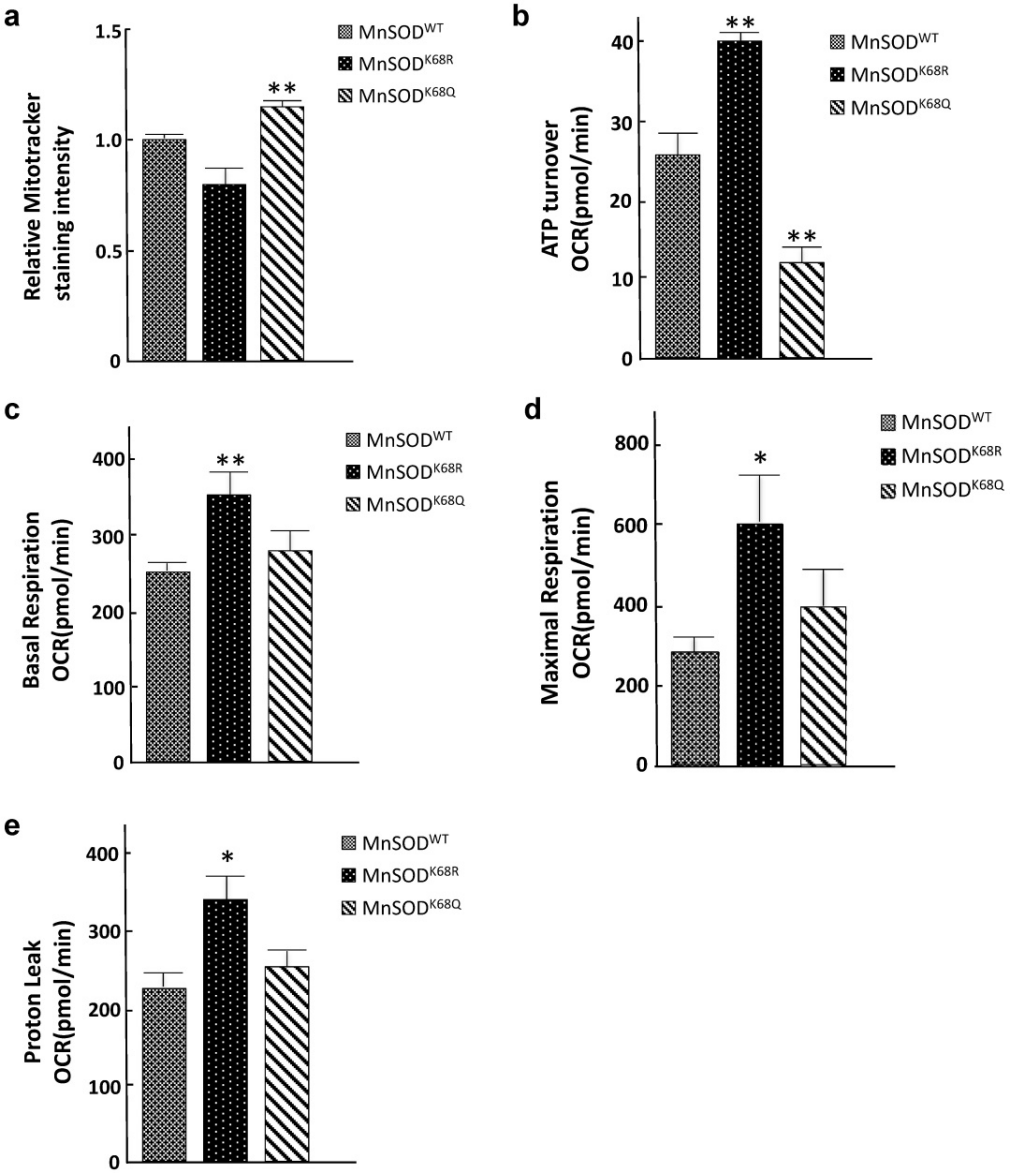
** Acetylation/deacetylation status in MnSOD-K68 regulates mitochondrial metabolism. (a)** One million of SOD^-/-^ MEFs overexpressing *MnSOD^WT^*, *MnSOD^K68R^*, and *MnSOD^K68Q^* were stained with 100nM MitoTracker Green (Cell Signaling) solution diluted in 1X PBS and analyzed using FITC channel via flow cytometry. Stained cell populations were further analyzed using FlowJo; **(b)** SOD^-/-^ MEFs overexpressing *MnSOD^WT^*, *MnSOD^K68R^*, and *MnSOD^K68Q^* were measured for ATP turnover rate determined by the difference between oxygen consumption rate (OCR) before and after adding ATP synthesis inhibitor oligomycin; **(c)** Basal respiration OCR was measured by the difference between OCR before and after adding complex I/III inhibitor rotenone/antimycin mixture; **(d)** Maximal respiration OCR was determined by the difference between OCR before and after adding mitochondria uncoupler CCCP/FCCP; **(e)** Proton leak rate was measured by the difference between the OCR after adding oligomycin and after adding rotenone/antimycin. All experiments were done in triplicates. Error bar represents ±1 SEM. *p<0.05, and **p<0.01. Three groups were analyzed using a one-way ANOVA test via GraphPad Prism 6.

Since mitochondria are central to oxidative respiration and metabolism, it seems logical to assume that structure alterations, due to increased MnSOD-K68-Ac, would alter mitochondrial function, as well as overall cellular metabolism. To address this idea, we examined several parameters associated with mitochondrial metabolism to explain the phenotypes observed from the electron microscopy above. Mitotracker Green FM assays were performed to measure mitochondrial mass/abundance among established SOD^-/-^ MEFs expressing *MnSOD^WT^*, *MnSOD^K68R^*, and *MnSOD^K68Q^*. The total mitochondria mass was found to increase in SOD^-/-^ MEFs expressing *MnSOD^K68Q^*, as compared to controls expressing either *MnSOD^WT^* or *MnSOD^K68R^* (Fig. 4a). Due to the significant decrease in mitochondrial size observed among MEFs infected with *MnSOD^K68Q^*, the detected rise in mitochondrial abundance may be interpreted as a compensatory mechanism for the cells. We subsequently examined the mitochondrial respiratory parameters of these cell lines using Seahorse XF24 extracellular flux analyzer. Upon the addition of ATP synthesis inhibitor oligomycin, ATP turnover rate for SOD^-/-^ MEFs expressing *MnSOD^K68Q^* was significantly lower than control SOD^-/-^ MEFs expressing *MnSOD^WT^* or *MnSOD^K68R^* (Fig. 4b), indicating that *MnSOD^K68Q^* expression likely disrupted oxidative phosphorylation within the mitochondria, which agreed with the loss of organized cristae structure alignment shown above. In addition, other factors affecting mitochondrial respiration, including basal respiration (Fig. 4c), maximal respiration (Fig. 4d), and proton leak (Fig. 4e), were significantly increased in SOD^-/-^ MEFs expressing *MnSOD^K68R^*, as compared to control cells expressing either *MnSOD^WT^* or *MnSOD^K68Q^*. This increase indicates that the acetylation/deacetylation balance of MnSOD-K68, not solely its acetylation, is critical for cellular function and could alter mitochondrial metabolism. These results strongly suggest that MnSOD acetylation not only affects superoxide detoxification but also disrupts mitochondrial function and morphology.

### MnSOD-K68-Ac shows elevated peroxidase activity when supplemented with iron

Our lab has provided evidence that MnSOD-K68-Ac exhibited significantly reduced superoxide dismutase enzymatic activity and increased peroxidase activity, which contributes to a tumor-permissive phenotype 30. Previous research has also indicated that under very specific physiological conditions, in addition to Mn^2+^_,_ the incorporation of iron (Fe^2+^) appears to modify MnSOD enzymatic activity 41. For example, iron incorporation into MnSOD disrupts the physiological oxidation/reduction, or redox potential of the enzyme's active site and inhibits superoxide detoxification 42. In fact, the incorporation of iron into MnSOD *in vivo*, under specific conditions, has appeared to inhibit superoxide detoxification 41. Moreover, MnSOD from *Escherichia coli* is present as a mixture of manganese- and iron-bound forms 41; however, manganese binding to MnSOD is favored under cellular conditions of oxidative stress, whereas Fe^2+^ binding is increased under anaerobic conditions 42, 43.

**Figure 5:**
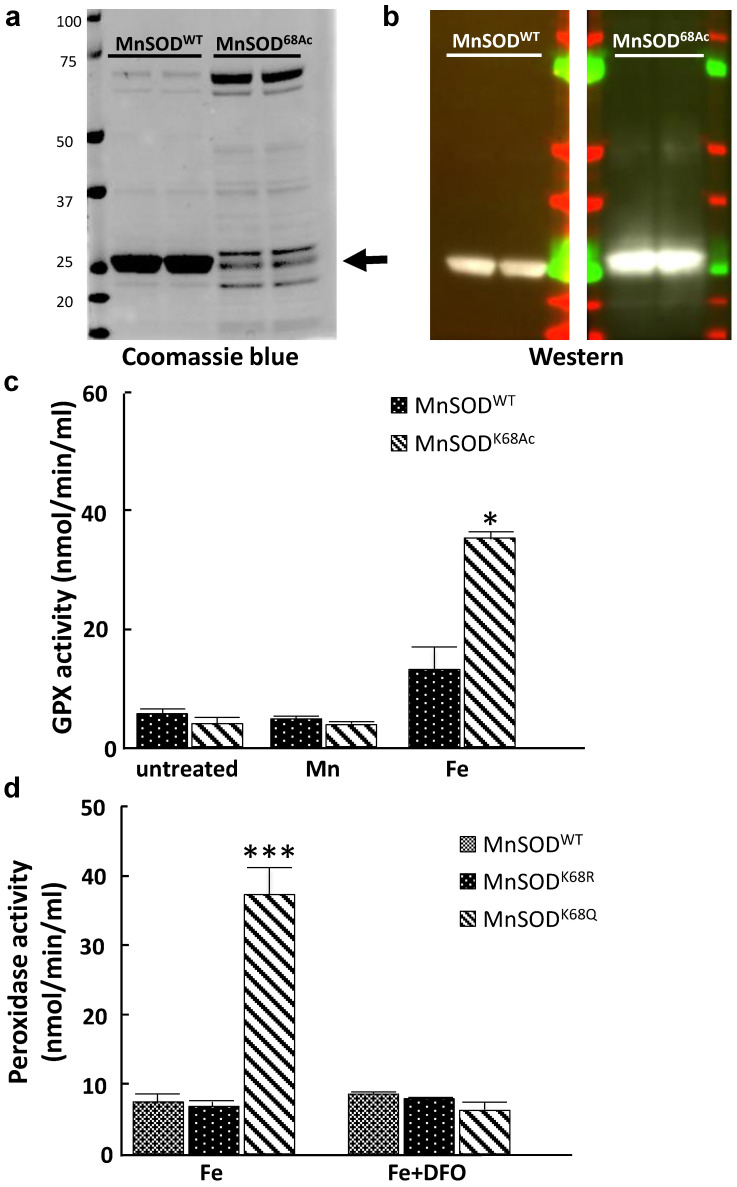
** MnSOD-K68-Ac exhibits higher peroxidase activity when incorporated with iron. (a.b)** The expression of purified MnSOD-WT and MnSOD-K68-Ac protein was verified by Coomassie blue **(a)** staining (as arrow pointed) and immunoblotting **(b)** for MnSOD and MnSOD-K68-Ac. **(c)** 10 µg purified protein of MnSOD-WT or MnSOD-K68-Ac was loaded with 200 µM Mn^2+^, or 200 µM Fe^2+^ was measured for enzymatic peroxidase activity. **(d)** pMEF cells were transfected with FLAG-MnSOD^WT^, FLAG-MnSOD^K68R^, or FLAG-MnSOD^K68Q^, and treated with 200 µM Fe^2+^ with or without 25µM deferoxamine (DFO) cell lysates were incubated with anti-FLAG beads. Pulled-down MnSOD^WT^, MnSOD^K68R^ and MnSOD^K68Q^ were measured for enzymatic peroxidase activity. Representative images are shown. All experiments were done in triplicates. Error bar represents ±1 SEM. *p<0.05, ***p<0.001.

Here, we further explored whether the iron addition to the MnSOD-K68 acetylated form would direct its peroxidase enzymatic activity. Consequently, we utilized a recombinant bacterial protein purification system where we co-transformed pEVOL-AcKRS, expressing an acetyl-lysyl-tRNA synthetase/tRNA^CUA^ and pET21a-MnSOD^K68TAG^ into bacteria. This model system results in the site-specific incorporation of an acetyl motif onto the lysine at amino acid 68 in MnSOD. Successful protein production and purification was confirmed by both coomassie blue staining (Fig. 5a) and immunoblotting with antibodies against MnSOD and MnSOD-K68-Ac (Fig. 5b).

These purified proteins, MnSOD-WT and MnSOD-K68-Ac were supplemented with either 200 µM Mn^2+^ or 200 µM Fe^2+^. In these experiments, no change in GPX activity was observed with either MnSOD-WT or MnSOD-K68-Ac, with or without the addition of Mn^2+^ (Fig. 5c, middle two bars), as compared to MnSOD-WT (left two bars). In contrast, the addition of Fe^2+^ increased GPX activity in MnSOD-K68-Ac (right two bars). To extend these results, we used a tissue cell culture system to verify if Fe^2+^ is necessary for peroxidase activity in MnSOD isolated from mammalian cells. We transfected pMEF and 293T cells with *FLAG-MnSOD^WT^*, *FLAG-MnSOD^K68R^* (deacetylation mimic mutant), or *FLAG-MnSOD^K68Q^* (acetylation mimic mutant), and then treated infected cells with 200 µM Fe^2+^, with or without 25µM deferoxamine (DFO), which inhibits the iron ion from binding. Results showed that pulled-down MnSOD-K68Q exhibited a significant increase in peroxidase activity, compared to MnSOD-WT or MnSOD-K68R (Fig. 5d, Fig S5, left three bars). Lastly, the addition of DFO reversed the increase in peroxidase activity in the presence of Fe^2+^, strongly suggesting that MnSOD-K68-Ac peroxidase activity is significantly enhanced with the addition of iron.

### MnSOD-K68-Ac alters the molecular dynamics and charge distribution of MnSOD

While several MnSOD acetylation sites 25-29 modulate MnSOD activity, we focused on K68 acetylation, which is triggered by nutrient conditions known to link mitochondrial metabolism with *in vivo* phenotypes from: (i) caloric restriction 20, (ii) time restricted fasting 19, and (iii) exercise 27, 28. We recently discovered a potential mechanism by which K68-Ac directs MnSOD structure or functional activity. Specifically, we showed that acetylation of MnSOD lysine 68 destabilizes and/or prevents the maintenance of tetrameric MnSOD 30. In addition, we detected that the monomeric form of MnSOD is enriched due to K68-Ac and, more importantly, displays a peroxidase activity, which is required for both its tumor promoter and tumor cell resistance properties. However, the underlying molecular protein dynamics that destabilize and/or prevent the maintenance of tetrameric MnSOD remains unknown. To address this, we worked with the Northwestern University Structural Biology Core (NUSBC) to compare the molecular dynamics among MnSOD-K68-Ac, MnSOD-K68Q, and MnSOD-WT.

**Figure 6:**
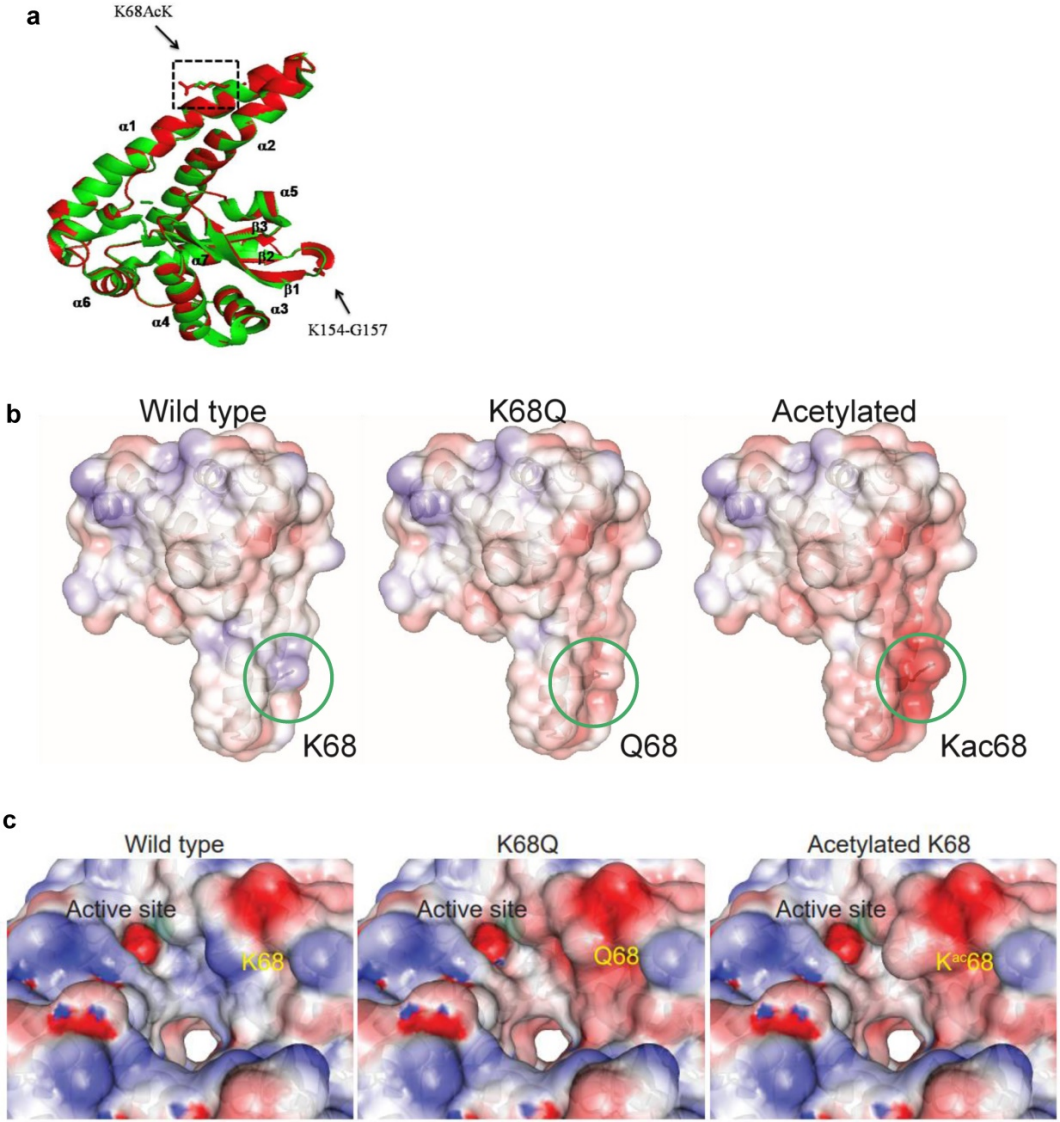
** Molecular dynamics of MnSOD upon its acetylation status. (a)** The MnSOD-K68 acetylation locates at the α1/α2 helical structure of the MnSOD monomer. **(b)** Molecular dynamic simulation shows the charge distribution at K68 during its WT state, acetylation state or acetylation mimetic (MnSOD^K68Q^). Blue color indicates positive charge distribution and red color indicates negative charge distribution. **(c)** A close-up view of the charge distribution and structural change around MnSOD-K68 is displayed. The green ball indicates the Mn^2+^ location.

Protein molecular dynamics simulations of tetrameric MnSOD done by NUSBC revealed that the acetylation of K68 happens along the α1/α2 helices of each monomer of MnSOD (Fig. 6a). Overall, there is a decrease in the positive surface charge distribution (area in red represents a decrease in positive protein surface charge) along the α1/α2 helices at the K68 location for MnSOD-K68-Ac and MnSOD-K68Q (Fig. 6b). If looked closer, a more obvious difference in the decrease in positive surface charge and the increase in bulk volume adjacent to the Mn^2+^ (Mn^2+^ is shown as a green ball) binding site/tetramerization interface of MnSOD-K68-Ac can be observed, as compared to MnSOD-WT (Fig. 6c). A nearly identical change in the α1/α2 helix surface charge was also observed for MnSOD-K68Q (Fig. 6c, middle panel). These results are of importance since Mn^2+^ interacts with a hydroxyl group, leading to a relatively negative charge that forms an electrostatic bond with the positive surface of the α1/α2 helix, suggesting that K68-Ac decreases the stability of the electrostatic bond between the Mn^2+^-hydroxyl group and the α1/α2 protein surface. This change in overall charge near the Mn^2+^ may be the underlying reason why Fe^2+^ can easily replace Mn^2+^. While these results are limited to molecular simulations, they do further confirm our genetic and biochemical data 30, 31 and provide an intriguing potential mechanism accounting for how K68-Ac may lead to **(i)** enrichment of monomeric MnSOD; **(ii)** loss of superoxide detoxification activity; and **(iii)** gain of a peroxidase activity.

## Discussion

MnSOD had been established and recognized as a tumor suppressor primarily due to its enzymatic role in the clearance of superoxide accumulation and detoxification of the mitochondria 44, 45. While MnSOD functions as a tumor suppressor; once tumorigenesis occurs, clinical data also suggest that MnSOD levels correlate with more aggressive human tumors, implying a potential dual function of MnSOD in the regulation of metabolism 34, 46. Our group 30, 31, 47, along with others 25, 26, highlighted the importance of the post-translational modification in MnSOD enzymatic activity and its effect on *in vivo* phenotypes, as regulated by SIRT3, that directs mitochondrial protein acetylation status. It has been widely published that *Sirt3* silencing leads to aberrant accumulation of ROS upon stress, which induces genome instability, mitochondrial reprogramming, and the disruption of the normal physiology of cellular energetics 20-24, 27-29, 48. SIRT3 functions as a mitochondrial TS where, when *Sirt3* has been genetically deleted, a tumor-permissive phenotype has been observed using both *in vitro* assays that measure transformation and tumorigenesis *using in vivo* murine models. Clinical observations also suggested a decrease in SIRT3 expression in breast cancer specimens 20. Our lab has found that decreased SIRT3 expression, and increased MnSOD acetylation, is correlated with poor clinical prognosis in human breast cancer and specifically associated with luminal B breast tumor malignancies, which share similar tumor characteristics with those of the mammary tumors observed in mice lacking *Sirt3* 7, 49.

In addition, we have identified earlier a specific subgroup of women with luminal B breast malignancies, an ER+ tumor subtype that displays aggressive characteristics including high Ki-67, high tumor grade, poor prognosis, and resistance to endocrine therapy, which exhibit a significant increase in MnSOD-K68-Ac, as compared to luminal A tumors. We have also recently uncovered that tumor cells that exhibit a significant increase in MnSOD-K68-Ac promote a tamoxifen-resistant phenotype *in vitro* and potentially directs the formation of a monomeric form of MnSOD, which functions as a peroxidase. In this regard, we suggest that the increased expression of MnSOD in the later stages of the multifactorial process of carcinogenesis might be explained by the disrupted equilibrium between acetylated and deacetylated MnSOD, and the resulting imbalance between the corresponding monomeric and tetrameric forms. In this paper, we further explored the possibilities of how MnSOD-K68-Ac would disrupt the mitochondria structure and function, due at least in part to metabolic stress, and whether this MnSOD-K68-Ac signature can be extended and applied to a broader therapy-resistant aggressive breast cancer model.

Metabolic stress, specifically the aberrant accumulation of ROS inside mitochondria, is an early event in tumorigenesis and an established hallmark of cancer 50, 51. ROS can accumulate as a byproduct of endogenous processes, exogenous conditions, and/or therapeutic agents 38, 52. In this regard, it is becoming increasingly clear that MnSOD is a major target that directs mitochondrial physiology and metabolism. Several recent reviews 53-55 have discussed and summarized that metabolic reprogramming may cause the activation of DNA repair, upregulation of anti-apoptotic pathways or immunosuppression of tumor microenvironment, which could explain the endocrine therapy and chemotherapy resistance observed in cancer recurrence. However, the exact mechanism behind this observation remains unclear. Here, we proposed the possibility of chemotherapy resistance induced by aberrant mitochondrial metabolism through the regulation of MnSOD-K68 acetylation status. We showed that MnSOD-K68-Ac promotes a transformation-permissive phenotype, and this signature can indeed be extended to a chemotherapy-resistant breast cancer cell model in a dose-dependent manner using cisplatin and doxorubicin. Moreover, expression of the acetylation mimetic *MnSOD^K68Q^* significantly disrupts mitochondrial ultrastructure and metabolism, which is a well-recognized phenotype commonly observed upon tumorigenic progression. Therefore, it is reasonable to suggest that MnSOD-K68-Ac is an important post-translational modification target that leads to the reprogramming of mitochondrial metabolism in cancer, which may be responsible for chemotherapy and endocrine therapy resistance.

Lastly, using computerized simulations of MnSOD structure, we were able to clearly visualize the charge distribution of MnSOD around K68, which is located at a critical position adjacent to the active site, as well as affecting the Mn binding site. We also provided data on the potential function of iron cooperation that directs the MnSOD enzymatic function towards a peroxidase. Thus, does this switch toward peroxidase activity when binding with iron damaged cells potentially lead to a tumor-permissive or more aggressive tumor phenotype? On this subject, there are several manuscripts 56-58, and this review 59, suggesting that iron overload is pro-tumorigenic. It was recently found that MnSOD can switch into a prooxidant peroxidase in manganese-deficient cells and mice by binding iron to the catalytic site 60. In addition, it is well established that prooxidants are oncogenic, and this idea has been suggested for iron usage and/or accumulation, MnSOD, and tumorigenesis 61. While our work does not address this idea, it does add to a growing list of literature investigating altered iron metabolism, mitochondrial detoxification enzymes, including MnSOD, and oncogenicity.

In summary, our results further provided a mechanism for how MnSOD-K68-Ac plays a role in cancer and suggested mitochondrial metabolism could be a potential target for ER+ breast cancer patients with this signature. We also broadened the applicability of the resistance directed by MnSOD acetylation to standardized therapy used in treating the more aggressive subtypes of breast cancer. While further research can be done in animal models and within clinical sample settings, this study can serve as groundwork for identifying MnSOD as a crucial mitochondrial regulating enzyme in carcinogenesis upon its acetylation/deacetylation status.

## Supplementary Material

Supplementary figures.
