# Supplementary material for: MnSOD Lysine 68 acetylation leads to cisplatin and doxorubicin resistance due to aberrant mitochondrial metabolism
Source: Int J Biol Sci. 2021 Mar 19;17(5):1203–16. doi: 10.7150/ijbs.51184 (PMC8040469; doi:10.7150/ijbs.51184)
Supplement: Supplementary file 1 — Supplementary figures. [file ijbsv17p1203s1.pdf]

Gao et al
Figure. S1

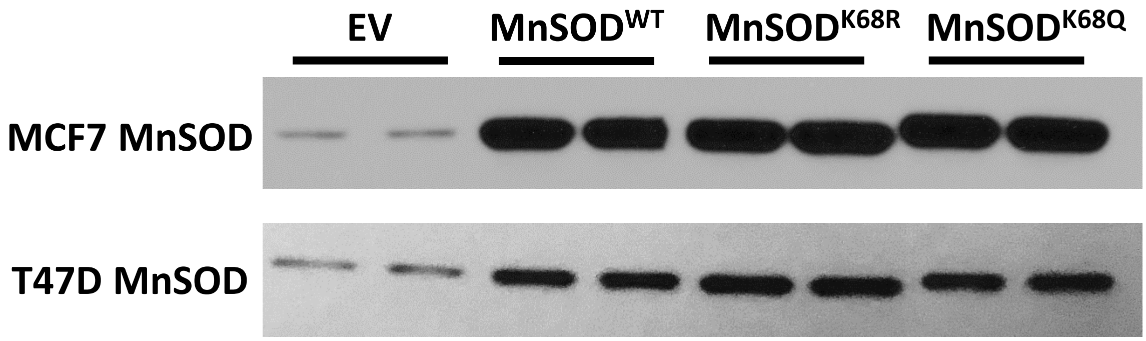

Figure. S1 Protein expression of MnSOD in MCF7 and T47D cells infected with empty vector, MnSOD^{WT}, MnSOD^{K68R}, andMnSOD^{K68Q}

MCF7 and T47D cells were lentivirally infected with empty vector, MnSOD^{WT}, MnSOD^{K68R} and MnSOD^{K68Q} . The cell lines were selected and cultured with medium with puromycin and the protein expression of MnSOD in those cell lines was detected through western blot.

Gao et al
Figure. S2

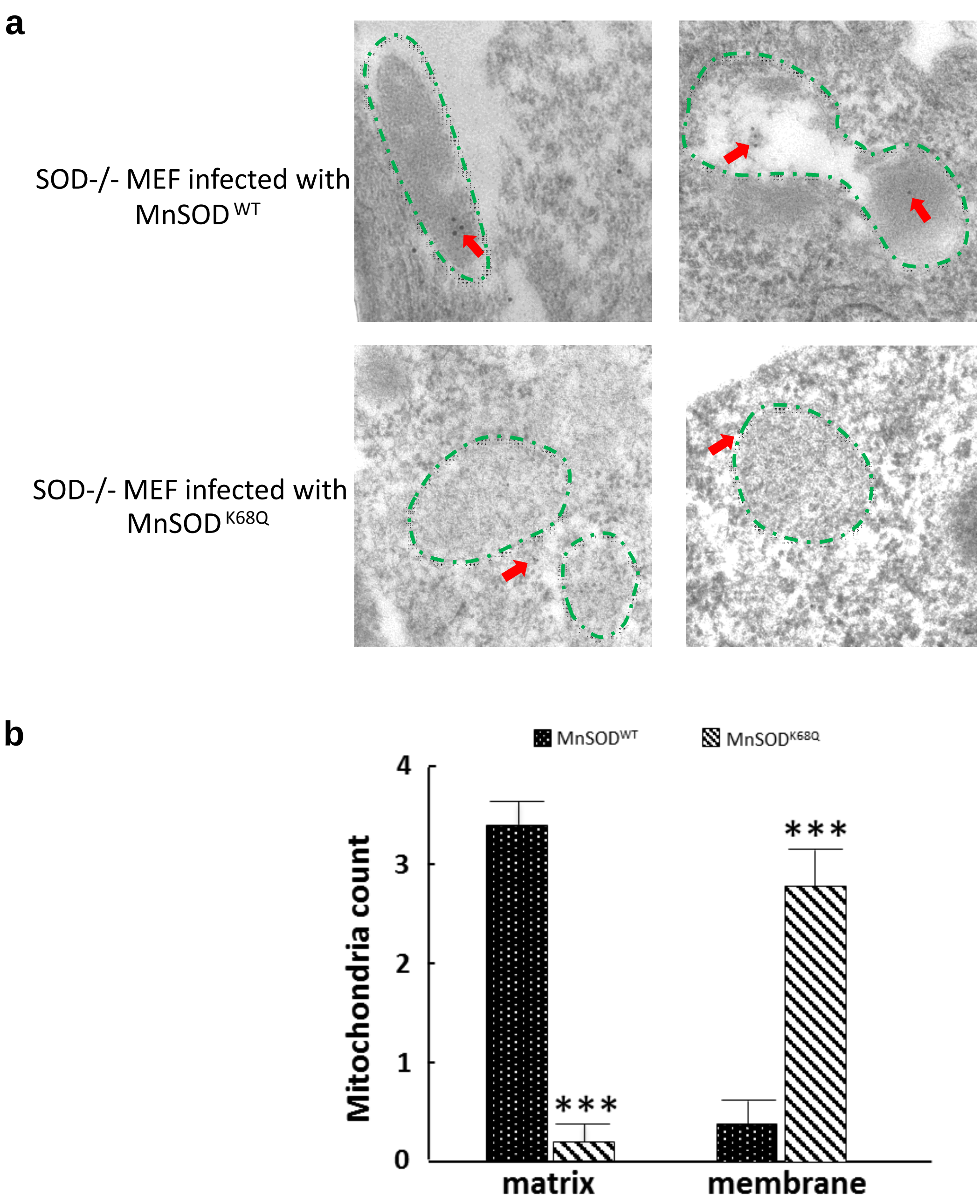

Figure. S2 Immunogold electron microscopy of SOD$^{-/-}$ MEF infected with MnSOD^{WT} or MnSOD^{K68Q}

(a) Localization of MnSOD was identified within the mitochondria using immunogold electron microscopy where the protein of interest was immunoblotted with primary antibody of MnSOD and then was bound with gold particles. The location of the particles was visualized under a FEI Tecnai Spirit G2 transmission electron microscope. **(b)** The distribution of the gold particles either in the mitochondrial matrix or on the mitochondrial membrane were quantified.

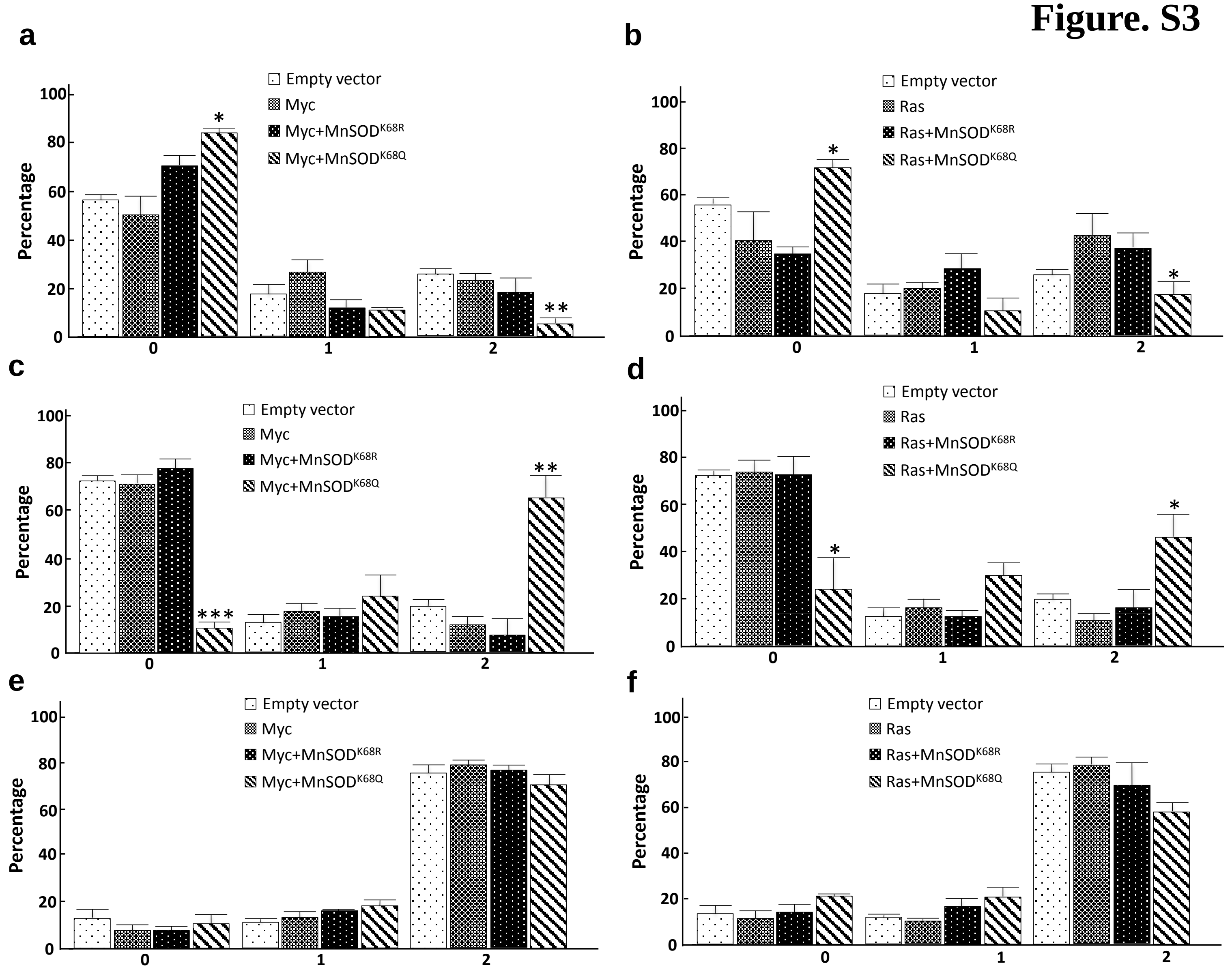

Figure. S3 Mitochondrial size, cristae structure, and electron density level of mitochondria between pMEF cells infected with empty vector, Myc or Ras, Myc or Ras with MnSOD^{K68R}, and Myc or Ras with MnSOD^{K68Q}
(a,b) Mitochondrial size of the pMEF cells infected with Myc or Ras with MnSOD^{K68R} or MnSOD^{K68Q} is quantified as described (0=small<0.7um, 1=intermediate, 2=large>0.8um) and visualized via bar graph. **(c,d)** Cristae structure clarity is quantified and visualized as described visualized (0=clear, 1=intermediate, 2=destroyed). **(e,f)** Quantification (0=low, 1=intermediate, 2=high) of electron density in mitochondria of pMEF cells was performed and visualized via bar graph. All experiments were done in triplicates. Error bar represents ±1 SEM. *$p<0.05$, **$p<0.01$.

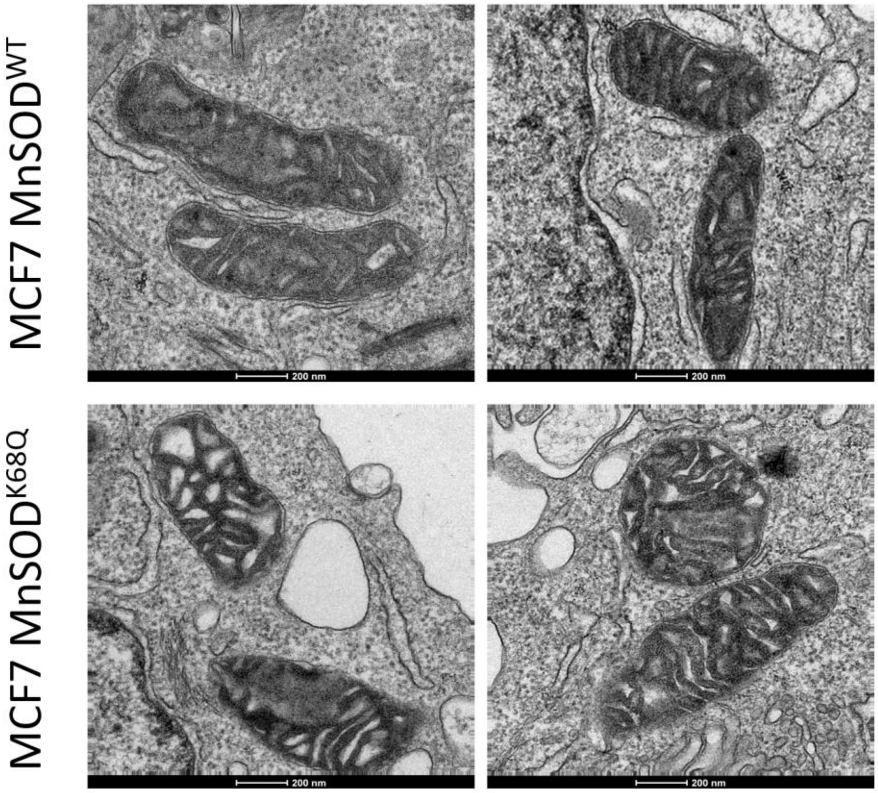

Figure.S4 Mitochondria ultrastructure of breast cancer MCF7 cells was visualized via transmission electron microscopy

MCF7 cells infected with either $MnSOD^{WT}$ and $MnSOD^{K68Q}$ were collected and fixed for embedding and sectioning (see Methods section). Samples were imaged using a FEI Tecnai Spirit G2 transmission electron microscope. Representative images are shown as above.

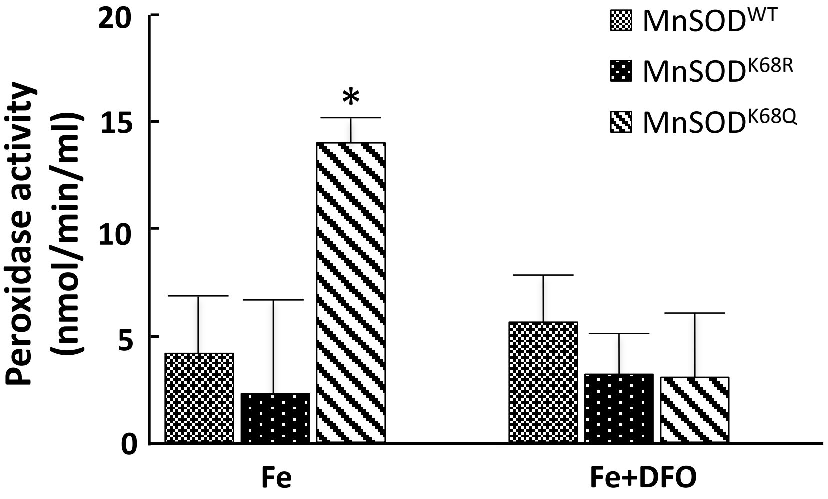

Figure. S5 Measured enzymatic peroxidase activity of pulled-down MnSOD from 293T cells

293T cells were transfected with FLAG tagged $MnSOD^{WT}$, $MnSOD^{K68R}$, $MnSOD^{K68Q}$ and then pulled down with anti-FLAG beads. Subsequently, the pulled down $MnSOD^{WT}$, $MnSOD^{K68R}$, $MnSOD^{K68Q}$ were measured for peroxidase activity with the addition of Fe and the addition of Fe with the iron chelator deferoxamine.
